# IL-4Rα signalling influences behavioural and immune responses in Trypanosoma brucei-infected mice: Evidence for iloprost as a neuroprotective agent

**DOI:** 10.1016/j.ibneur.2025.08.014

**Published:** 2025-08-19

**Authors:** OS Olaolu, H. Davids, GB Dealtry, A.S. Abubakar, ET Obishakin, B. Iliyasu, IA Azeez, DC Ajonijebu

**Affiliations:** a Department of Human Physiology, Faculty of Science, Nelson Mandela University, Gqeberha, South Africa; b Biotechnology Centre, National Veterinary Research Institute, Vom, Nigeria; c Nigerian Institute for Trypanosomiasis Research, Vom, Nigeria; d Department of Veterinary Anatomy, Faculty of Veterinary medicine, University of Jos, Nigeria; e Department of Human Anatomy and Physiology, Faculty of Health Sciences, University of Johannesburg, South Africa

**Keywords:** IL-4Rα┴, Trypanosomiasis, Neuroinflammation, Anxiety, Iloprost

## Abstract

This study investigated the effects of *Trypanosoma brucei* infection on neuroinflammation, immune response, and behaviour in both wild-type (WT) and IL-4Rα inhibited (IL-4Rα**┴**) mice. To achieve this, 9-week-old WT and IL-4Rα**┴** mice were infected with *T. brucei* intraperitoneally (5 ×10^2^ parasites) and the treated groups received 200 μg/kg/day of Iloprost intraperitoneally. Results from infected animals showed that behavioural activity and inflammation were reduced in animals treated with Iloprost. Euthanasia was performed on 12 days post-infection (dpi), and prefrontal cortex (PFC), Hippocampus (HPC) and blood were collected. PCR confirmed the presence of *T. brucei* in the brain and blood, demonstrating its ability to cross the blood-brain barrier. CXCL10, a key chemokine implicated in neuroinflammation, was elevated in infected mice, particularly in IL-4Rα**┴** mice, which lack the ability to initiate protective type 2 immune responses. Treatment with Iloprost suppressed CXCL10 expression and reduced inflammation. Behavioural assessments revealed that *T. brucei* infection induced anxiety-like behaviours and hypoactivity. Interestingly, IL-4Rα inhibition appeared to reduce anxiety-like behaviours in infected mice, while Iloprost treatment had an anxiolytic effect. Locomotor deficits were observed in infected mice, with IL-4Rα**┴** mice showing more pronounced hypoactivity. However, both Iloprost and Diminazine improved locomotor activity. At the molecular level, IL-4Rα inhibition resulted in upregulation of pro-inflammatory cytokines such as TNF-α and elevated nitric oxide levels, contributing to CNS inflammation. Iloprost treatment reduced these markers and supported anti-inflammatory pathways. These findings highlight the complex interplay between immune regulation, neuroinflammation, and behaviour in trypanosome infection, with IL-4Rα signalling playing a critical role in modulating disease outcomes. Therapeutic interventions targeting these pathways, such as Iloprost, may offer neuroprotective benefits in African trypanosomiasis.

## 1 Introduction

Trypanosomiasis, caused by protozoan parasites of the genus *Trypanosoma*, is known to induce neuroinflammation, which can significantly affect host behaviour. In particular, infections with *Trypanosoma brucei* have been associated with neuropsychiatric manifestations, including anxiety, depression, and memory impairment, especially during the chronic stages of the disease (Torres et al., 2022). These behavioural changes are believed to arise from a complex interplay of social, psychological, and biological stressors, notably persistent neuroinflammation and oxidative stress within the central nervous system (CNS) (Joseli Lannes-Vieira et al., 2023).

The immune system plays a crucial role in modulating neuroinflammation and subsequent behavioural outcomes. Cytokines, which are signalling proteins released by immune cells, can influence CNS function (Dantzer et al., 2008). Pro-inflammatory cytokines like interleukin-1 beta (IL-1β) and tumor necrosis factor-alpha (TNF-α) have been implicated in promoting anxiety-like behaviours (Gosselin and Rivest, 2007, McAfoose and Baune, 2009, Wang and Shuaib, 2002, Rossi et al., 2012). Conversely, anti-inflammatory cytokines such as interleukin-4 (IL-4) are associated with anxiolytic (anxiety-reducing) effects (Moon et al., 2011, Derecki et al., 2010). For instance, IL-4 knockout mice, which lack this anti-inflammatory cytokine, display increased anxiety-like behaviours, suggesting that IL-4 plays a protective role against anxiety (Moon et al., 2015).

IL-4 exerts its effects through the interleukin-4 receptor alpha (IL-4Rα). Inhibition of IL-4Rα can disrupt IL-4 signalling, potentially leading to an imbalance between pro- and anti-inflammatory responses in the brain (Quagliato and Nardi, 2017). This imbalance may exacerbate neuroinflammation and contribute to anxiety-related behaviours (Zhou et al., 2002). Research indicates that blocking IL-4Rα prevents the anxiolytic effects of certain treatments, highlighting the importance of IL-4 signalling in regulating anxiety (Li et al., 2019).

In the context of trypanosome-induced neuroinflammation, inhibiting IL-4Rα could potentially worsen anxiety-related behaviours by amplifying inflammatory responses within the CNS. Understanding the interplay between trypanosome infections, neuroinflammation, and IL-4 signalling pathways is crucial for developing therapeutic strategies aimed at mitigating anxiety and other neuropsychiatric manifestations associated with parasitic infections.

## 2 Materials and methods

### 2.1 Ethical considerations and animal husbandry

Ethical approval for this study was obtained from the Animal Ethics Committee of the National Veterinary Research Institute (NVRI), Nigeria (Ref: AEC/02/102/21), and the Research Ethics Committee of Nelson Mandela University, South Africa (Ref: A20-SCI-PHY-001). All experimental procedures complied with the guidelines outlined in the NIH Guide for the Care and Use of Laboratory Animals (2011, Ref: N01-OD-4–2139).

A total of sixty-four male Swiss albino mice (9 weeks old) were used for the study. The animals were obtained and maintained at the research animal facility of the Nigerian Institute for Trypanosomiasis Research (NITR). Mice were housed in groups of four per cage under standardized environmental conditions, including 23–25^0^C room temperature, 50 ± 5 % relative humidity, and a 12:12 h light/dark cycle (lights on from 06:00–18:00). Animals had ad libitum access to a standard laboratory rodent diet and water.

Swiss albino mice were selected due to their widespread use in biomedical research, particularly in pharmacological and infectious disease studies, including models of trypanosomiasis. Their genetic homogeneity contributes to reduced inter-individual variability and improved reproducibility of results. The Trypanosoma brucei parasite used in this study was maintained through serial passage in Swiss albino mice.

### 2.2 Animal grouping and treatment

Following one week of acclimatization, mice were randomized to two primary treatment groups (n = 32 per group): IL-4Rα-inhibited (IL-4Rα┴) and wild-type (WT). IL-4Rα┴ mice received a single intraperitoneal (*i.p*) injection of Dupilumab (0.5 mg/kg; BioVision Inc., Canada), an IL-4Rα antagonist that inhibits IL-4 and IL-13 signalling pathways (Tomkinson et al., 2001). WT mice received a subcutaneous injection of PBS.

Each primary group was further subdivided into four subgroups (n = 8), with three subgroups per treatment arm infected intraperitoneally with 5.0 × 10² *Trypanosoma brucei* parasites (Federe strain) (McCarroll et al., 2015), while one subgroup remained uninfected and served as negative controls.

At four days post-infection (dpi), one infected subgroup from each treatment group received *i.p* injection of Iloprost (200 µg/kg/day, Abcam, South Africa) for four consecutive days, which commenced at the onset of established parasitaemia. The dosing regimen and timing were selected based on previous in vivo study demonstrating Iloprost’s anti-inflammatory and vascular protective properties during immune responses (Uchiyama-Tsuyuki et al., 1995).

The remaining infected subgroups received either a single curative dose of Diminazene aceturate (4 mg/kg, i.p.) or PBS. Diminazene aceturate was used as a reference treatment to assess therapeutic efficacy, given its established trypanocidal activity and routine application in experimental models of trypanosomiasis. The selected dose has been shown toeffectively clear parasitaemia without inducing toxicity (Eckersall et al., 2001), facilitating comparative evaluation between full parasite clearance (Diminazine -treated), immune compromised (Iloprost-treated), and untreated controls (PBS).

### 2.3 Parasitic invasion of the CNS

To ascertain parasitic invasion of the CNS parenchyma, detection of the Trypanosoma ITS gene was performed on day 12 (D12) post-infection using conventional PCR. Amin et al. (2008) demonstrated that, at this stage of infection, parasites are no longer restricted to the cerebral blood vessels but have begun infiltrating the parenchyma. Similarly, Frevert et al. (2012), using a murine model and intravital brain imaging, showed that bloodstream forms of *T. b. brucei* and *T. b. rhodesiense* can enter the brain parenchyma within hours, before substantial microvascular inflammation becomes evident. These findings suggest that while PCR alone cannot distinguish between vascular and parenchymal presence, the detection of parasite DNA in brain tissue at 12 dpi is consistent with prior histological evidence supporting both vascular and parenchymal localization. At the same time, the concentration of CXCL-10, a candidate biomarker for late-stage HAT, was measured in the brains of T. brucei-infected mice using ELISA (Amin et al., 2009).

### 2.4 Detection of ITS gene

For amplification of *T.brucei* DNA, the Internal Transcribed Spacer (ITS)-1 forward primer (5′-CCGGAAGTTCACCGATATTG-3′) and ITS-1 reverse primer (5′-TGCTGCGTTCTTCAACGAA-3′) were used, as designed by Njiru et al. (2011). PCR was performed in a 25 μL reaction volume consisting of 10 ng template DNA, 2.5 μL 10 × FastStart High-Fidelity Reaction buffer (Roche, Mannheim, Germany), 15 mM MgCl_2_, 200 mM dNTPs, and 0.2 mM each of forward and reverse primers (Inqaba, Nigeria). The thermocycling conditions (GeneAmp PCR System 9700, Applied Biosystems, Singapore) were as follows: 94°C for 5 min; 35 cycles of 95°C for 30 sec, 59°C for 30 sec, and 72°C for 45 sec; and a final extension step at 72°C for 10 min. A negative control, no template control was included in each PCR experiment. After amplification the PCR products were separated by 1.5 % agarose gel electrophoresis. 1.5 g of agarose powder was added to a beaker with 100 mL 1x Tris-Borate-EDTA (TBE) buffer. The mixture was heated in a microwave until the agarose fully dissolved and the solution was clear (about 1–2 min, checking and swirling every 20 sec). The solution was allowed to cool to around 50–55°C, then 10 μL ethidium bromide was added and mixed. The agarose solution was poured into a gel casting tray with a 10 well-comb in place to create wells, and it was allowed to solidify for 20–30 min. Once solidified, the comb was gently removed and the gel was placed in the electrophoresis chamber with 1x TBE buffer, ready for sample loading and electrophoresis. 10 μL 100 bp molecular ladder (New England, Inqaba) in the first well and 15 μL samples were added to each well. Electrophoresis (Bio-rad® Powerpac) was run at 100 V for 45 min. With an aid of gel documentation (Bio-rad Gel-Doc™ XR+) with Image lab™ software, the image was visualized.

### 2.5 Determination of CXCL-10 in the brain

CXCL-10 determines the ability of parasite to cross the BBB, as it plays a key role in immune cell recruitment and inflammation (Masocha et al., 2008, Rodgers et al., 2019). The PFC and HPC were homogenized and the supernatant were used to determine the concentrations of C-X-C motif chemokine ligand CXCL-10 by ELISA using murine CXCL-10 (CXCL-10, cat. E-EL-M0032) standard ELISA Development kit (Elabscience USA), according to the manufacturer’s instructions.

### 2.6 Assessment of body weight changes

Body weight was determined by placing each mouse on a weighing balance (Marte scientific, Germany) cleaned with 70 % alcohol between animals. The body weight was measured every week during the experiment.

### 2.7 Behavioural tests

#### 2.7.1 Elevated plus maze test

Anxiety-like behaviour was assessed on D9 using the elevated plus maze (EPM) test (Vollert et al., 2014). The maze was made from medium-density wood fibre with a matte black acrylic surface and consisted of four arms: two open (without walls) and two enclosed by 30 cm high walls (50 ×10 cm). The EPM is a widely used behavioural assay for rodents, validated to assess anxiety-related behaviours and the effects of pharmacological agents, as well as identify the brain regions and mechanisms involved.

One hour before testing, mice were transferred to the experimental room in their home cages to acclimatize. During testing, mice were placed at the junction of the four arms, facing an open arm, and allowed 5 min to explore. Parameters recorded included the number of entries and time spent in each arm (open vs. closed) and head dips. An increase in entries and/or time spent in the open arms is indicative of reduced anxiety-like behaviour. Between trials, the maze was cleaned thoroughly with 70 % ethanol to eliminate all olfactory cues.

#### 2.7.2 Open field test

Open Field Test (OFT) is a commonly used model of locomotion and anxiety-like behaviour established to assess emotionality in mice (Kraeuter et al., 2018). This test uses a camera to measure the movement of the test mice in the peripheral and central zones of a 42 × 42 × 42 cm polyvinyl chloride box. On D9, OFT was conducted. It is based on exposing an animal to an unknown environment from which it cannot escape because of surrounding barriers. The mice were put in the middle rectangular device and many behavioural characteristics typically horizontal/vertical activity, time spent in the central square, rearing, grooming and resting, mice prefer not to stay in the middle, illuminated part of the apparatus and prefer to wander close to the walls (thigmotaxis) (Gogas et al., 2007) were recorded during a defined period of 5 min. These observations indicate the exploratory, locomotory and anxiety-like behaviours of mice. Between tests, the maze was cleaned thoroughly with 70 % ethanol to eliminate all olfactory cues. These behavioural characteristics were calculated 3 times for each animal per behaviour.

### 2.8 Animal sacrifice and brain collection

On D12, mice were euthanized with 100 % halothane applied to a cotton wool placed in a glass chamber with a cover. Samples were collected at 12 dpi to capture the critical transition phase of *Trypanosoma brucei brucei* infection in the brain parenchyma. Importantly, Frevert et al. (2012), using a murine model with intravital brain imaging, demonstrated that bloodstream forms of *T. b. brucei* and *T. b. rhodesiense* can penetrate the brain parenchyma within hours, at a stage when significant microvascular inflammation has not yet developed. Extravascular bloodstream forms were viable as indicated by motility and cell division, and remained detectable for at least 3 days post-infection, suggesting the potential for parasite survival in the brain parenchyma. Thus, 12 dpi represents a window in which neuropathological changes (neuroinflammation), vascular alterations, and host immune signalling can be investigated, enabling a clearer assessment of disease progression and potential therapeutic effects. Each animal was kept in the chamber for 3–4 mins until loss of consciousness and reflexes. Afterward, about 0.5 mL of whole blood was collected via cardiac puncture into a heparinized vacutainer. The whole brain was then removed using appropriate bone forceps, sectioned into two halves, and the prefrontal cortex and hippocampus were dissected out from the left hemisphere. The brain tissues were snap-frozen in liquid nitrogen and stored at −80°C in a bio-freezer until needed for molecular analysis. The right hemisphere was cryopreserved or fixed in 10 % neutral buffered formalin (pH 7.2, NaH_2_PO_4_, Na_2_HPO_4_ and 10 mL formalin + 90 mL distilled water) histological analysis.

### 2.9 Histological technique

After halothane anaesthesia, mouse brains (cerebrum and cerebellum) were separated from the skull and the right hemisphere fixed in buffered formalin (10 %), processed in ethanol and xylene (as described below) then embedded in paraffin wax and sectioned to obtain 4 mm paraffin sections with a microtome (Reichert-Jung, Biocut). According to Drury and Wallington (1980), sections were stained with hematoxylin and eosin. The tissues were fixed in 10 % buffered formalin (AC Scientific®) (10 mL formaldehyde, 4 g NaH_2_PO_4_, 6.5 g Na_2_HPO_4_) for at least 7 days for effective fixation of the tissues. Since most commonly employed fixatives are aqueous, the next step in tissue processing is usually that of dehydration and “clearing” (removal of all water from the specimen). Graded solutions of ethanol (Absolute Alcohol Neon® 70 %-100 %) were used for this purpose for 30 min each. These were changed frequently to maintain their desiccating properties. A variety of clearing agents are available, but the most common are xylene and limonene derivatives. Xylene I and II were used as clearing agents for 15 min each. Afterwards the tissue was placed in a metallic casting rack and filled with hot paraffin for 2 h and allowed to cast. With the use of a microtome, 4 mm sections were obtained from the tissue. Once the tissue was affixed to glass slides, paraffin was removed before the staining procedure. This was accomplished by placing the sections in a carrier, heating them to 56 °C for at least one hour (preferably longer—to evaporate water from the glass slides and further anchor the tissue), and immersing them for 3–5 min in each of two successive containers of xylene. Most histologic dyes penetrate tissue best if it has been re-hydrated; thus, the passage of the slides through containers of graded ethanol solutions (absolute; 95 %; 70 %) and distilled water is necessary before staining. The importance of proper paraffin clearance cannot be overemphasized. If a sizable quantity of wax remains in the sections, dyes will not be able to penetrate and impregnate constituent tissues. The regressive method of staining was adopted, which refers to a technique where tissue purposefully over-impregnated with hematoxylin and then modified by decolourization with dilute hydrochloric acid. Haematoxylin staining was for 1 min 30 sec and then the section was washed with water before eosin was added for 30 sec and washed as before, finally a coverslip was used to cover the stained-glass slide.

### 2.10 Haematological analysis

To evaluate the impact of *T. brucei* infection on the haematological profile, 0.2 mL whole blood was collected in a microtube containing an anticoagulant ethylenediaminetetraacetic acid (EDTA). Total white blood cell count (WBC), lymphocyte count in percentage analyses were performed using the automated haematology analyser (Mindray BC-2800Vet, Germany). The principle is based on the electrical impedance for cell counting and a cyanide-free technique for measuring haemoglobin in each of the samples. The machine processes each sample by aspirating 200 μL whole blood and in 4–5 min, results for each parameter were obtained.

### 2.11 Brain sample collection, DNA methylation and quantitative PCR

On D12, mice were euthanized with 100 % halothane applied to a cotton wool placed in a glass chamber with a cover. Each animal was kept in the chamber for 3–4 mins until loss of consciousness and reflexes. The mice were sacrificed. After removing the brains, prefrontal cortical tissues and hippocampus were dissected out on ice followed by RNA extraction (Norgen Biotek, Canada) followed by two-step RT-PCR, the iScript™ cDNA synthesis kit (Bio-Rad™, USA) was used. They were performed according to the manufacturer’s instruction. cDNA was subjected to quantitative real-time (qPCR) using the following primers in Table 1. DNA extraction (ZR Genomic DNA-Tissue Mini Prep; Inqaba biotec, South Africa) and bisulfite conversion (EZ DNA methylation kit; Zymo Research, USA), were performed according to the manufacturer’s instructions. Methylated DNA was then subjected to qPCR on Light Cycler 2.0 (Roche Diagnostics (Pty) Ltd., South Africa) using the following primers in Table 2. The PCR reaction mixture consisted of 5 μL DNA template, 10 μL SYBR Green I (Roche Diagnostics, South Africa), 2 μL each of 15 μM primer pair stock and 1 μL DNA-free water. CT values were chosen within the linear range while differences in methylation between samples were determined using the comparative CT method as previously described Schmittgen and Livak, (2008).

**Table 1:** List of Primer Sequence for mRNA Synthesis.

**Target genes**	**Primers**	**Primer Sequence (5ˈ-3ˈ)**	**Primer length**	**Annealing Temp**	**Reference Paper**
GAPDH	Forward Reverse	GTGGCAAAGTGGAGATTGTTG ACCAGTAGACTCCACGACATA	21 21	60 °C	Modarresi et al., 2021
IL−4R	Forward Reverse	TGACCTCACAGGAACCCAGGC GAACAGGCAAAACAACGGGAT	21 21	60 °C	Dewals et al., 2010
BDNF	Forward Reverse	TCACAGCGGCAGATAAAAAG TCAGTTGGCCTTTGGATACC	20 20	60 °C	Zheng et al., 2011
TNF-α	Forward Reverse	GCCTCTTCTCATTCCTGCTTG CTGATGAGAGGGAGGCCATT	21 20	60 °C	Scarneo et al., 2018

**Table 2:** List of Primer sequences for DNA methylation synthesis.

**Target gene**	**Primer**	**Primer sequence (5ˈ-3ˈ)**	**Primer length**	**Annealing Temp**	**Reference paper**
GAPDH	Forward Reverse	GCCAAAAGGGTCATCATCTCCGC GGATGACCTGCCCACAGCCTTG	23 22	60 °C	Ajonijebu et al., 2018
IL−4	Forward Reverse	TCCATGCACCGAGATGTTTGTACC CGTAGGATGCTCCCTTTATGAACG	24 24	60 °C	Costelloe et al., 2008
IL−13	Forward Reverse	GGAGCTGAGCAACATCACACA GGTCCTGTAGATGGCATTGCA	21 21	60 °C	Overbergh et al., 2003
IL−4Rα	Forward Reverse	TGACCTCACAGGAACCCAGGC GAACAGGCAAAACAACGGGAT	21 21	60 °C	Dewals et al., 2010
BDNF	Forward Reverse	TCACAGCGGCAGATAAAAAG TCAGTTGGCCTTTGGATACC	20 20	60 °C	Modarrasi et al., 2021

Additionally, the PFC and HPC were homogenized, and the supernatant was used to determine concentrations of cytokines TNF, Nitric oxide and NF-κB by a solid-phase sandwich ELISA assay. The assay was performed using ELISA kits (Elabscience USA) with plates pre-coated with antibodies for tissue necrosis factor-alpha (TNF-α, cat E-EL-M0049), nuclear factor-kappa B (NF-kB, cat. E-EL-M0031) and nitric oxide (NO, cat. E-BC-K035-M) according to the manufacturer’s instructions.

### 2.12 Statistical analysis

GraphPad Prism software (version 10) was used for the statistical analysis. At first, we tested all data for Gaussian distribution using Shapiro-Wilk normality test. Where distribution was normal, data were analysed using two-way analysis of variance followed by Tukey comparison post-hoc test. All data are expressed as the mean ± SD with level of significance set at *P* < 0.05.

## 3 Results

### 3.1 Detection of ITS in the brain

**Fig. 1:**
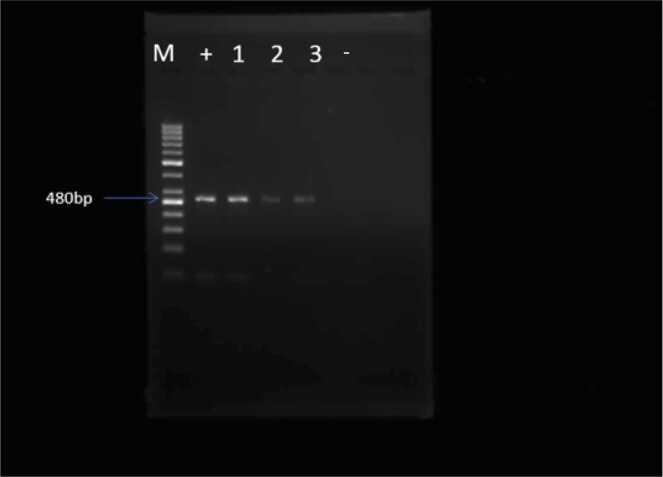
Agar Gel documentation of *T. brucei* in the brain parenchyma amplified at 480 bp using the ITS gene. M= 100 bp molecular ladder, + =positive control (the stock parasite 3.98 ×10^6^/mL), -=negative control, 1 =brain IL-4Rα┴ infected, 2 = brain WT infected, 3 =blood IL-4Rα┴.

A previous study by Frevert et al. (2012) demonstrated that African trypanosomes invade the murine brain parenchyma during the early stages of infection, preceding the establishment of meningoencephalitis. Using the intravital brain imaging, the authors showed that bloodstream forms of T.b brucei and T.b rhodesiense penetrate the brain parenchyma within hours post-infection, prior to the development of significant microvascular inflammation. Resting on this knowledge, the present study employed conventional PCR to detect and quantify the ITS gene in the brains of *T.b* brucei-infected mice at 12dpi. The parasite was successfully detected in the brain at this time point, as evidenced by a PCR amplicon of 480 bp. (Fig. 1). Early detection of the parasites in the brain supports the hypothesis that *T. brucei* may access the CNS via alternative routes, such as the choroid plexus and circumventricular organs, as previously suggested (Frevert et al., 2012).

### 3.2 Elevated CXCL 10 level in cortical and hippocampal brain regions following trypanosome infection

**Fig. 2:**
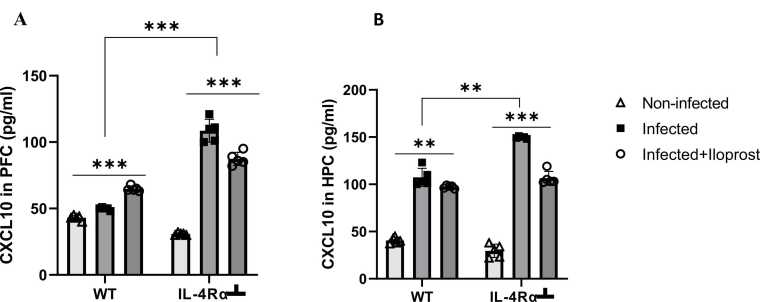
CXCL-10 in the PFC (A) and HPC (B) of mice infected with *T.brucei* and the uninfected controls. Data represented as mean ± SD of independent experiments. Statistical significance was determined using Two-Way ANOVA with Tukey’s post hoc test. (n = 5, **= p < 0.005, ***=p < 0.001).

Previous studies have shown that CXCL-10 is one of the key chemokines that facilitates the penetration of *T. brucei* into the brain parenchyma (Amin et al., 2009). The PFC and HPC of infected and non-infected WT and IL-4R┴ mice were therefore examined for endogenous CXCL-10 production using sandwich ELISA. There was a significant (p < 0.001) increase in the CXCL-10 levels in the infected compared to the non-infected WT mice in both PFC and HPC (Fig. 2A and B). The CXCL-10 levels in IL-4R-infected mice were significantly increased compared to the non-infected IL-4R┴ in both PFC and HPC to a greater level than the WT group (Fig. 2A and B). This suggests that *T. brucei* are more likely to have crossed the BBB with CXCL-10 levels increased in both PFC and HPC, more in the IL-4R┴-infected mice. In the PFC, Iloprost-treated infected WT mice had increased CXCL-10 compared to uninfected and infected WT mice. IL-4R┴ mice had increased CXCL-10 in infected and infected plus Iloprost compared to uninfected, but the Iloprost-treated infected mice had less CXCL-10 than the infected mice without Iloprost. But IL-4R┴ mice had higher levels of CXCL-10 following infection and infection plus Iloprost than WT

In the HPC both WT and IL-4R┴ had elevated CXCL-10 following infection and decreased CXCL-10 after Iloprost treatment compared to untreated infected mice (Fig. 2B).

### 3.3 Body weight changes in mice post-infection

**Fig. 3:**
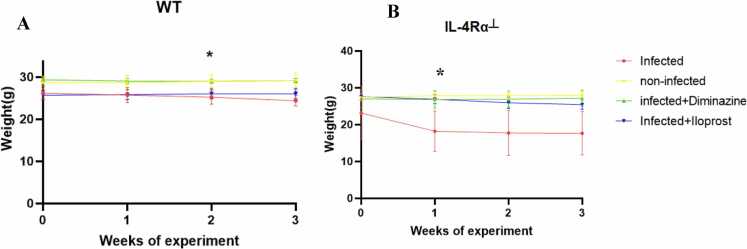
Effects of Diminazine and Iloprost treatments on T.b-induced body weight changes in Wild-type (A) and IL-4Rα┴ (B) mice. The results are presented as mean ± SD. Statistical significance was determined using one-way ANOVA (n = 5; *p ≤ 0.05 ** p ≤ 0.005).

Body weight was measured at baseline during the one-week acclimatisation period (prior to *T. brucei* infection and treatment), as well as throughout the treatment phase and post-treatment at weekly intervals (weeks 1–3). There was a significant decrease in body weight of the untreated WT mice at week 2 and of the IL-4Rα┴ mice at week 1 post-infection (Fig. 3A and B). This indicates that trypanosome infection in IL-4Rα┴ infected mice in week 1 could be severe due to the inhibition of IL-4R (immunocompromised system) and also trypanosome infection. In the WT mice, weight reduction in week 2 could be due to the infection.

### 3.4 Impact of *T. brucei* infection on WBC populations and neutrophil-lymphocyte ratio

The study initially investigated the effects of *T. brucei* infection on the white blood cell population and further examined its impact on the neutrophil-lymphocyte ratio as a biomarker for trypanosome-induced neuroinflammation.

**Fig. 4:**
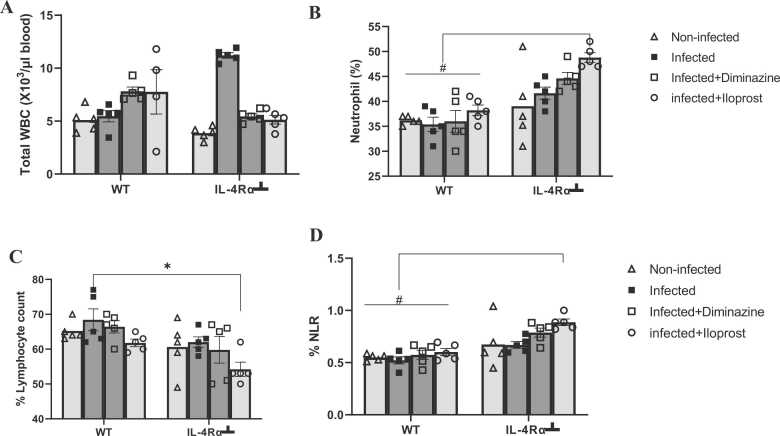
Effects of *T.brucei* infection and Iloprost treatment on (A) White blood cell count, (B) Neutrophil count, (C) Lymphocyte count, and (D) Neutrophil-Lymphocyte ratio. The results are presented as mean ± SD (n = 5). Statistical significance was determined using 2-way ANOVA followed by the Tukey *post hoc* test of multiple comparison (* p ≤ 0.05 vs WT; # p ≤ 0.005 vs WT).

As shown in Fig. 4A, there was no significant difference in white blood cell count, across all treatment groups (p > 0.05). However, the IL-4Rα-infected mice exhibited the highest count for the WBC, although not significant, this could be due to the inhibition of IL-4R and the burden of infection. The Neutrophil counts were slightly lower across all WT groups, compared to the IL-4Rα┴ Iloprost-treated mice. However, WT Iloprost and Diminazine-treated mice had higher total WBC compared to the infected untreated mice. The percentage of neutrophils of the WT-infected was significantly lower (P < 0.05) compared to the IL-4Rα┴ Iloprost-treated group (Fig. 4B). The Lymphocyte counts in the IL-4Rα┴ infected mice decreased significantly (p < 0.05) following Iloprost treatment, compared to the WT mice (Fig. 4C). Fig. 4D shows the neutrophil-lymphocyte ratio, where Iloprost-treated IL-4Rα┴ mice exhibited significantly decreased values (p < 0.05) compared to the values of the WT group.

### 3.5 IL-4Rα inhibition enhanced anxiolytic-like exploratory behaviour in trypanosoma infected mice

This study investigated the behavioural changes in *T.brucei*-infected mice, one of which was EPM. Anxiety-like exploratory behaviours provided insights into the interplay between infection-induced condition and behavioural outcomes.

**Fig. 5:**
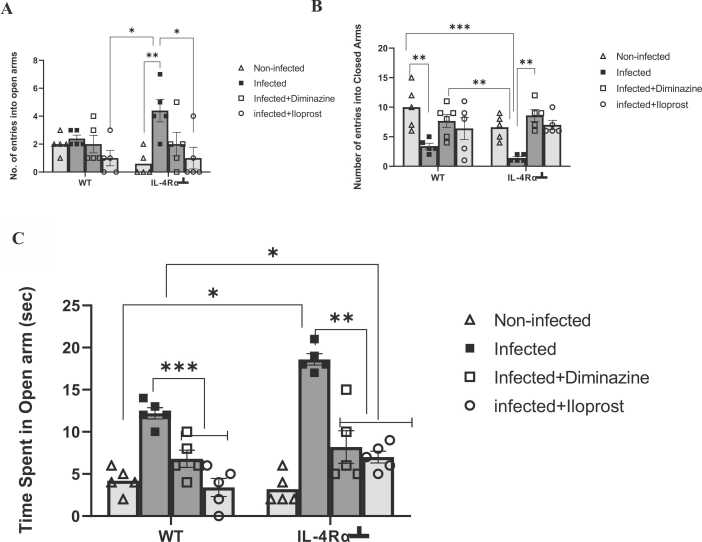
Effects of *T.brucei* infection and Iloprost treatment on anxiety-related fuctions of WT and IL-4Rα┴ mice tested in the elevated plus maze. The results are presented as mean ± SD (n = 5). Statistical significance was determined using two-way ANOVA followed by the Tukey *post hoc* test of multiple comparisons (n = 8 *p ≤ 0.05, ** p ≤ 0.01, *** p ≤ 0.001).

The EPM test revealed that the infected IL-4Rα┴ mice explored the open arms more frequently than other treatment groups, while the non-infected IL-4Rα┴ made fewer entries into the open arm (p < 0.05). In contrast, the infected WT mice treated with Iloprost entered the open arms less often (Fig. 5A).

There was a significant difference in the number of entries into the closed arm (P < 0.001). WT non-infected mice explored the closed arms more frequently compared to the rest of the groups, while IL-4Rα┴ infected mice made fewer entries into the closed arm (Fig. 5B). Consequently, the time spent in the open arm was significantly different, with the IL-4Rα┴ group spending more time on the open arm than the WT group of mice, which suggests that inhibition of IL-4R promoted a fearless/less anxious response than the WT. The interaction between factors was also significant (*F*3,32 = 4.725, *P* = 0.007) (Fig. 5C). There was no significant difference observed in the number of head dips, which indicates awareness of height (Fig. 5D).

### 3.6 Diminazine and iloprost treatments enhanced motor activity and anxiety-related functional components in trypanosome infection

This study further investigated the locomotory and exploratory changes of *T.brucei*-infected mice, using an open field test.

**Fig. 6:**
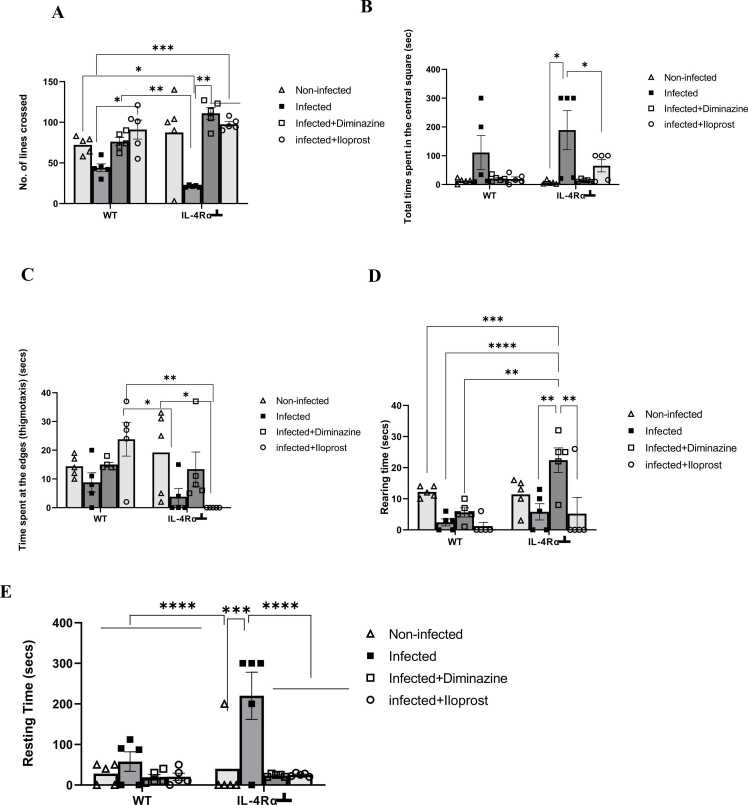
Effects of *T.brucei* infection and Iloprost treatment on locomotion and anxiety-related functions of WT and IL-4Rα┴ mice assessed in an open field. The results are presented as mean ± SD (n = 5). Statistical significance was determined using two-way ANOVA followed by the Tukey *post hoc* test of multiple comparisons (*p ≤ 0.05, ** p ≤ 0.005 *** p ≤ 0.01, **** p ≤ 0.001).

The OFT showed that the number of lines crossed by WT-infected mice was fewer than the non-infected mice. It was significantly(p < 0.005) fewer in IL-4Rα┴ infected mice than in non-infected mice. IL-4Rα┴ Diminazene-treated mice made significantly more (p < 0.001) line crossings compared to Iloprost-treated IL-4Rα┴ mice, whereas WT Iloprost-treated mice made significantly more line crossings compared to the infected mice and Diminazine-treated mice (*F*_3,32_ = 17.16, *P* < 0.001). In contrast, the most crossings were shown in Diminazine-treated IL-4Rα┴ mice (Fig. 6A), indicating increased locomotor activity and anxiety (p < 0.005). This was also the case with Iloprost-treated mice, but not as much as for Diminazine-treated mice. This indicates increased locomotory/ exploratory activities in WT Iloprost-treated mice and IL-4Rα┴ Diminazine-treated mice.

Next, the duration spent in the central square was analysed, focusing on this metric as an indicator of exploratory behaviour and anxiety levels. Infected mice spent the most time in the central square for both the WT and IL-4Rα┴ mice, compared to their non-infected counterpart although not statistically significant (*P* = 0.50). However, the interactions between treatment groups reveal statistically significant differences (*F*_3,32_ = 7.799, *P* = 0.0005) in IL-4Rα┴ between the non-infected and infected and Iloprost treated mice (Fig. 6B), which implies poor locomotory/exploratory activity and as well anxiety-like behaviours.

Next, thigmotaxis was examined, measuring the time spent near the walls of the box as an additional indicator of anxiety-like behaviour. This behaviour reflects the animal's preference for staying close to the periphery, a typical response to stress or heightened anxiety levels. WT infected mice exhibited less thigmotactic behaviour compared to the non-infected, while the same was observed in the IL-4Rα┴-infected mice, with significantly (p < 0.05) decreased thigmotaxis compared to the non-infected. WT infected Iloprost treated mice exhibited significantly (p < 0.05) increased thigmotaxis compared to the infected IL-4Rα┴ mice, while the infected IL-4Rα┴ Iloprost treated showed decreased/no thigmotaxis compared to other groups. The interaction between treatment groups and controls (infected and non-infected) for both WT and IL-4Rα┴ mice was significantly different (F_3,32_ = 4.44, *P* = 0.01) (Fig. 6C). This suggests that WT Iloprost-treated mice explored more than other treated groups.

The unsupported rearing time was then examined. This is a measure of vertical exploratory behaviour and an indicator of increased anxiety. Rearing time was notably observed in WT non-infected mice compared to the infected mice, reflecting their typical exploratory behaviour and locomotor activity. In the IL-4Rα┴ infected mice, there was decreased rearing time compared to non-infected mice. Diminazine-treated IL-4Rα┴ mice reared significantly (p < 0.005) more compared to the Iloprost-treated and infected IL-4Rα┴ mice (Fig. 6D). WT Iloprost-treated mice reared less compared to the Diminazine-treated mice. The interaction between factors was significant (F3,32 = 4.93, *P* = 0.006). Iloprost reduced rearing time for both IL-4Rα┴ and WT mice compared with the non-infected and infected groups (*P* < 0.05) (Fig. 6D).

Following this, resting time was evaluated, which serves as a measure of inactivity or immobility and can indicate behavioural responses such as fatigue, stress, or reduced motivation. Changes in resting time are often analyzed to assess the overall impact of experimental conditions on energy levels and anxiety-like states. WT-infected mice significantly (p < 0.0001) rested more compared to non-infected mice. Also, infected-IL-4Rα┴ significantly (p < 0.0001) rested more than the non-infected mice. Both Diminazine-treated and Iloprost-treated mice exhibited less resting time compared to the other groups (Fig. 6E).

### 3.7 Effects of trypanosome infection on the cerebrum

**Fig. 7:**
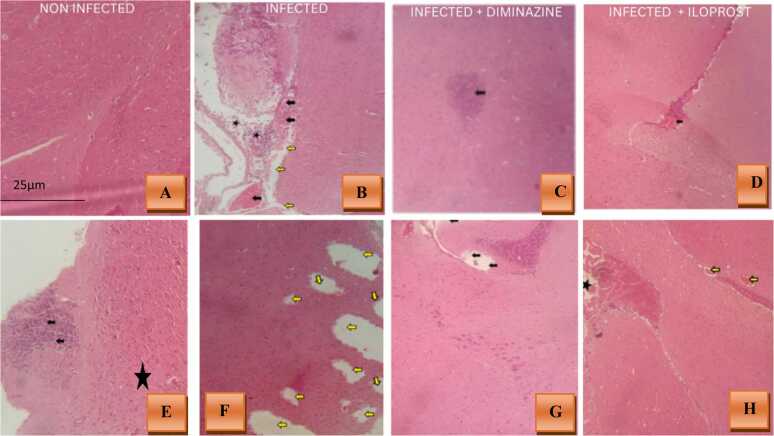
Histopathological staining using H & E. **(A)**WT non infected mice**(B)** WT Infected mice with no treatment showing necrosis (Yellow arrows), congestion of cerebral blood vessels (black arrows) with cellular infiltration (black stars), **(C)** WT Diminazine+infected mice showing cellular infiltration (black star) in the cerebrum, **(D)** WT Iloprost +infected mice showing congestion of cerebral blood vessel, (**E**) IL-4Rα┴ non-infected mice with cellular infiltration (black arrows) **(F)** IL-4Rα┴ Infected mice showing severe necrosis of the cerebral tissue (yellow arrows), **(G)** IL-4Rα┴ Diminazine +infection showing necrosis of the cerebral tissue (yellow arrows), **(H)** IL-4Rα┴ Iloprost+infection showing necrosis of cerebral tissue (Black arrows). Viewed under x40 objective.

Trypanosome infections are known to cause significant pathological changes in the brain, particularly in the cerebrum for all groups, due to the inflammatory and neurodegenerative effects of the parasite. Histological staining techniques provide valuable insights into these alterations by revealing structural damage such as necrosis, vascular congestion, and cellular infiltration. This study focuses on using histological staining to evaluate the extent and nature of cerebral tissue damage in trypanosome-infected mice, shedding light on the neuropathological impact of the infection. Histological examination of the cerebral cortex revealed distinct pathological changes across the WT and IL-4Rα┴ mice. In WT-infected mice, there was evidence of necrosis, congestion of cerebral blood vessels, and cellular infiltration (Fig. 7A). WT-Iloprost-treated mice displayed congestion of cerebral blood vessels, while WT-Diminazine-treated mice showed cellular infiltration in the cerebrum (Fig. 7B). IL-4Rα┴-infected mice exhibited severe necrosis of cerebral tissue, with IL-4Rα┴-Iloprost-treated and IL-4Rα┴-Diminazine-treated mice both showed necrosis of the cerebral tissue, highlighting varying degrees of neurodegeneration and vascular involvement in the cerebral cortex across the treatment groups (Fig. 7C, D, E and F).

### 3.8 Trypanosome induced transcriptional changes

Having determined the effective anxiogenic effect of Iloprost in WT and IL-4Rα┴ mice in the OFT and EPM during trypanosome infection (Fig. 5, Fig. 6), it may be that these behavioural changes result from transcriptional changes associated with *T. brucei* infection and/or signalling via the IL-4Rα chain. Therefore, this study investigates trypanosome-induced transcriptional changes, focusing on how host cells alter gene expression in response to trypanosome infection by analysing changes in the expression of the IL-4Rα, BDNF and TFN-α of both the HPC and PFC substrate regions of the brain.

#### 3.8.1 IL-4Rα

To further investigate the biological effects of the two sister molecules (IL-4 and IL-13), changes in the expression of IL-4Rα gene was examined., This receptor is known to mediate their shared signalling (Brombacher et al., 2017) in the PFC and HPC.

**Fig. 8:**
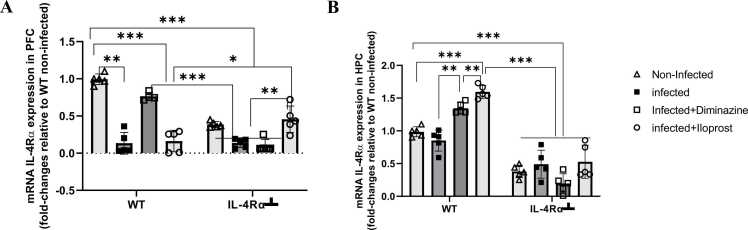
Effect of Iloprost on IL-4Rα mRNA expression in the PFC (A) and HPC (B) of mice infected with *T.brucei* and the control mice. Data represented as mean ± SD of independent experiments. Statistical significance was determined using Two-Way ANOVA with Tukey’s post hoc test. (n = 5 * = p < 0.05, **= p < 0.005, ***= p < 0.001).

In the PFC, there was a significant (p < 0.005) downregulation of IL-4Rα mRNA expression in WT infected mice compared to the WT non-infected mice. IL-4Rα┴ infected mice demonstrated downregulation of IL-4Rα mRNA expression compared to the non-infected (Fig. 8A). WT infected Diminazine-treated mice showed significant (p < 0.005) elevated IL-4Rα mRNA levels compared to WT non-infected, Iloprost-treated infected mice, and IL-4Rα┴ non-infected, infected and Iloprost-treated mice. Iloprost-treated IL-4Rα┴ mice also showed significantly (p < 0.005) elevated IL-4Rα mRNA levels compared to the Diminazine-treated, infected and non-infected IL-4Rα┴ mice (Fig. 8A).

The HPC was also examined. There was downregulation of IL-4Rα mRNA levels in WT infected mice compared to the non-infected mice and further downregulation in the IL-4Rα┴ infected and non-infected compared to the WT non-infected mice (Fig. 8B). There was a significant (p < 0.005) upregulation of IL-4R mRNA levels in the WT Iloprost-treated mice compared WT non-infected, Diminazine-treated and infected mice. There was significant downregulation of IL-4Rα mRNA levels (p < 0.05) in Diminazine-treated IL-4Rα┴ whereas upregulation in the WT compared to WT-noninfected mice. However, Iloprost and Diminazine-treated mice were upregulated compared to the WT non-infected mice in the hippocampus (Fig. 8B). This result also reveals that Iloprost-treated mice can induce the IL-4Rα expression in the hippocampus. The downregulation of IL-4Rα mRNA levels in the IL-4Rα┴ mice could be due to the inhibition of IL-4Rα

#### 3.8.2 BDNF

**Fig. 9:**
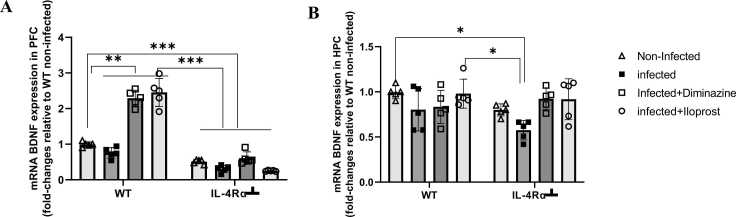
Effect of Iloprost on mRNA BDNF in the PFC (A) and HPC (B) of mice infected with *T.brucei* and the control mice. Data represented as mean ± SD of independent experiments. Statistical significance was determined using Two-Way ANOVA with Tukey’s post hoc test. (n = 5 *=p < 0.05, **=p < 0.005, ***=p < 0.001).

BDNF is a critical mediator of neuronal and immune-neuro interactions, which may be disrupted during trypanosome infection. The possible role of BDNF signalling pathways in the PFC and HPC during learning and reference memory phases was explored with WT and IL-4Rα┴ mice. WT infected mice had downregulation of BDNF mRNA in the PFC compared to non-infected mice. There was significant (p < 0.001) downregulation of BDNF mRNA levels in IL-4Rα┴ infected mice compared to WT non-infected mice. WT Iloprost-treated infected mice exhibited significant upregulation of BDNF mRNA levels compared to the non-infected and all IL-4Rα┴ mice in the PFC. WT Diminazine-treated infected mice also demonstrated significant (p < 0.005) upregulation of BDNF mRNA levels compared to the WT non-infected mice (Fig. 9B). Furthermore, during the novel memory phase of the task, the effects of steady expression levels of BDNF observed in the PFC (Fig. 9A) correlate to impaired reference memory in the infected mice (Fig. 9B).

In the HPC, there was downregulation of BDNF mRNA levels in the WT infected mice compared to WT non-infected mice. IL-4Rα┴ infected mice exhibited significant (p < 0.05) downregulation of BDNF mRNA levels compared to the WT non-infected mice. WT Iloprost-treated mice demonstrated significant (p < 0.05) upregulation of BDNF mRNA levels compared to the infected IL-4Rα┴ in the HPC (Fig. 9B) but in the PFC there was significant upregulation of BDNF expression in the infected and treated mice (Fig. 9A).

#### 3.8.3 TNF-α

**Fig. 10:**
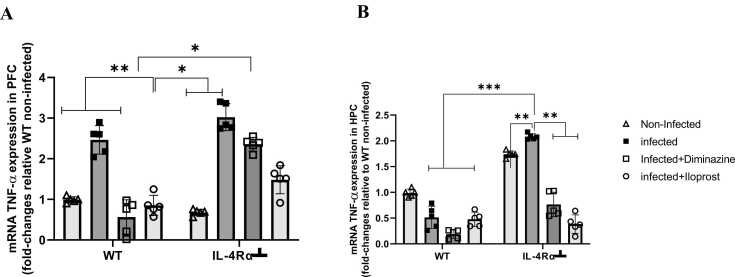
Effect of Iloprost on mRNA TNF-α in the PFC (A) and HPC (B) of mice infected with *T.brucei brucei* and their controls. Data represented as mean ± SD of independent experiments. Statistical significance was determined using Two-Way ANOVA with Tukey’s post hoc test. (n = 5 * = p < 0.05, **=p < 0.005 ***=p < 0.001).

Detection of TNF-α mRNA provides further evidence that this cytokine plays a key role in the development of inflammation in the CNS. A significant upregulation of TNF-α mRNA levels (p < 0.05) in the PFC of mice infected only with *T, brucei*, was found in both WT and IL-4Rα┴ groups compared to non-infected control mice (p < 0.05). However, in the infected WT mice, there was a downregulation of TNF-α mRNA expression in both the Diminazine- and Iloprost-treated groups, but not in IL-4Rα┴ mice, with no effect observed in the immune-compromised mice with IL-4Rα activity inhibited (p < 0.05, Fig. 10A).

Statistically, TNF-α gene expression in the HPC in all groups of infected WT mice did not differ significantly from the non-infected control mice (p > 0.05). However, TNF-α mRNA levels in the HPC of infected WT mice appeared to decrease (Fig. 10B). Further analysis revealed significant upregulation of hippocampal TNF-α mRNA in non-infected IL-4Rα┴ mice compared to their WT counterparts, although the levels were not as high as those in the IL-4Rα┴ infected mice (p < 0.05, Fig. 10B).

### 3.9 DNA methylation changes in the prefrontal cortex and hippocampus of trypanosome-infected mice

DNA methylation changes in the promoter regions of the IL-4, IL-13, IL-4Rα and BDNF genes in the brains of mice 12 days after *T.brucei* infection were assessed using bisulfite modification and qPCR techniques.

#### 3.9.1 Diminazine and iloprost treatments reversed trypanosome-induced IL-4 promoter hypermethylation in the prefrontal cortex of mice

**Fig. 11:**
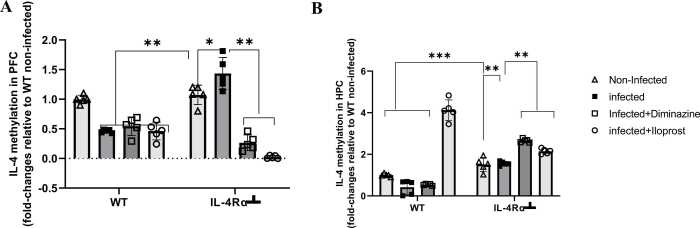
Expression levels of IL-4 in the PFC (A) and HPC (B) of mice infected with *T.brucei* and the control mice. Data represented as mean ± SD of independent experiments. Statistical significance was determined using Two-Way ANOVA with Tukey’s post hoc test. (n = 5, * = p < 0.05, **=p < 0.01, ***=p < 0.005).

After 12 days of *T.brucei* infection significant DNA hypomethylation of the *IL-4* gene promoter was detected in the PFC of all infected WT mice compared to non-infected control (p < 0.01, Fig. 11A). In contrast, IL-4Rα┴ mice infected with *T.brucei* exhibited significant DNA hypermethylation, indicating transcriptional downregulation of *IL-4* gene expression in this group, compared to the non-infected IL-4Rα┴ mice (p < 0.05, Fig. 11A). Interestingly, the altered methylation status in IL-4Rα┴ infected mice was reversed by both Diminazine and Iloprost treatments (p < 0.01, Fig. 11B).

#### 3.9.2 IL-13

**Fig. 12:**
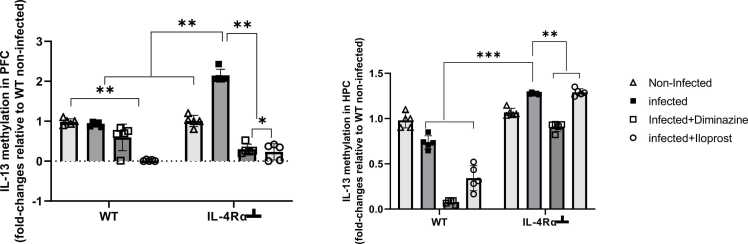
Expression levels of IL-13 in the PFC (A) and HPC (B) of mice infected with *T.brucei* and the control mice. Data represented as mean ± SD of independent experiments. Statistical significance was determined using Two-Way ANOVA with Tukey’s post hoc test. (n = 5, * = p < 0.05, **=p < 0.005, ***=p < 0.001).

Significant DNA hypomethylation of the *IL-13* gene promoter was observed in the PFC of all infected WT mice compared to non-infected control (p < 0.005, Fig. 12A). Conversely, IL-4Rα┴ infected mice exhibited significant DNA hypermethylation, indicating transcriptional downregulation of *IL-4* gene expression in this group, compared to the non-infected IL-4Rα┴ mice (p < 0.005, Fig. 12A). Notably, the altered methylation status in IL-4Rα┴ infected mice was reversed by both Diminazine and Iloprost treatments (p < 0.01, Fig. 12B). In the HPC, there was hypomethylation of the *IL-13* gene promoter in the WT-infected mice compared to the non-infected, while on the other hand, IL-4Rα┴ infected mice demonstrated significant hypermethylation of *IL-13*, indicating transcriptional downregulation. Iloprost-treated IL-4Rα┴ mice showed hypermethylation compared to the WT non-infected mice (p < 0.005, Fig, 12B). This also portrays the role of Iloprost and Diminazine in mitigating inflammation in these regions, except in the HPC for IL-4Rα┴ Iloprost-treated mice.

#### 3.9.3 Dimhinazine and iloprost treatments reversed trypanosome-induced IL-4Rα promoter hypermethylation in the prefrontal cortex of mice

**Fig. 13:**
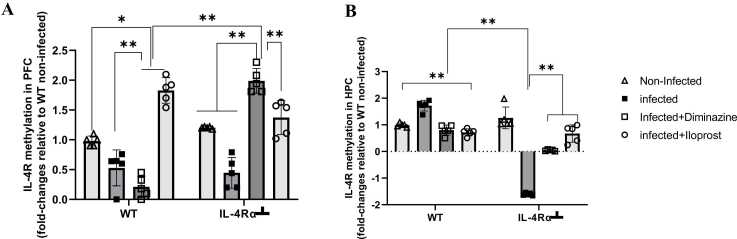
Expression levels of IL-4R in the PFC (A) and HPC (B) of mice infected with *T.brucei* and the control mice. Data represented as mean ± SD of independent experiments. Statistical significance was determined using Two-Way ANOVA with Tukey’s post hoc test. (n = 5, * = p < 0.05, **= p < 0.005).

DNA hypomethylation of the *IL-4Rα* gene promoter was detected in the PFC of infected WT mice compared to the non-infected mice. Similarly, IL-4Rα┴ infected mice exhibited hypomethylation of the *IL-4Rα* gene promoter compared to the non-infected. There was significant DNA hypermethylation in the promoter regions of *IL-4Rα* of Diminazine-treated WT and IL-4Rα┴ mice, (*P* < 0.05; *P* < 0.005, Fig. 13A) when compared to their respective controls. At the same time, Iloprost, showed significant hypermethylation in the WT and IL-4Rα┴ compared to non-infected mice (p < 0.005, Fig. 13A). This means that in the PFC, Iloprost treatment of infected IL-4Rα┴ mice led to epigenetic modification of the *IL-4Rα* gene. This epigenetic modification typically results in reduced expression of the *IL-4Rα* gene by hindering the binding of transcription factors to the DNA. In the HPC, there was significant (p < 0.005) hypermethylation of the *IL-4Rα* gene promoter in the infected WT mice compared to the non-infected mice. Conversely, infected IL-4Rα┴ mice exhibited significant hypomethylation of the *IL-4Rα* gene promoter compared to the non-infected mice (p < 0.005, Fig. 13B). This means trypanosome infection in the HPC of WT mice leads to an increase in methyl groups in the DNA sequence of *IL-4R* gene promoter. In the IL-4Rα┴ mice there is a decrease/decline in methylation levels at the *IL-4R* gene promoter or associated CpG islands which could result in the inhibition of the IL-4R and infection.

#### 3.9.4 Diminazine and iloprost treatments reversed trypanosome-induced BDNF gene promoter hypermethylation in the hippocampus of mice

**Fig. 14:**
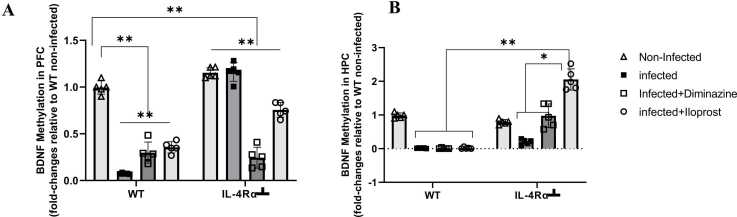
Expression levels of BDNF in the PFC (A) and HPC (B) of mice infected with *T.brucei* and the control mice. Data represented as mean ± SD of independent experiments. Statistical significance was determined using Two-Way ANOVA with Tukey’s post hoc test. (n = 5, * = p < 0.05, **=p < 0.005).

The epigenetic modification of the *BDNF* gene promoter was examined in the infected mice. In the PFC significant (p < 0.005) hypomethylation of the *BDNF* gene promoter was exhibited in all WT mice compared to the non-infected mice (Fig. 14A). However, in the IL-4Rα┴ non-infected mice there was significant hypermethylation of the BDNF gene promoter compared to the WT non-infected mice. All Iloprost and Diminazine-treated WT and IL-4Rα┴ showed hypomethylation of the *BDNF* gene promoter gene (p < 0.005, Fig. 14A). This will result in transcriptional downregulation of the *BDNF* gene. In the HPC, there was significant hypomethylation of the *BDNF* gene promoter in the infected, Diminazine and Iloprost-treated WT group compared to the non-infected mice, whereas IL-4Rα┴ non-infected mice showed hypomethylation compared to the WT non-infected mice. Diminazine-treated mice exhibited significant hypermethylation of the *BDNF* gene promoter compared to the WT non-infected mice (P < 0.005, Fig. 14B).

### 3.10 *T.brucei* enhanced neuroinflammatory changes in cortical and hippocampal regions of the brain

Inflammation associated changes in TNF, NF-κB, and NO in the were examined in the PFC and HPC of mice infected with *Tb* to assess the neuroinflammatory response associated with parasitic infection using ELISA. This analysis aimed to investigate the molecular mechanisms driving neuroinflammation and its potential impact on cognitive and behavioural dysfunctions in affected animals.

#### 3.10.1 Tumour necrosis factor-alpha (TNF-α)

**Fig. 15:**
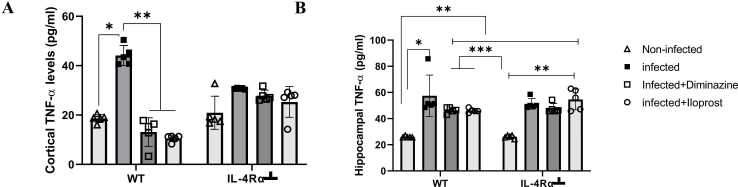
Assessment of TNF-α in the PFC (A) and HPC (B) of mice infected with *T.brucei* and the control mice. Data represented as mean ± SD of independent experiments. Statistical significance was determined using Two-Way ANOVA with Tukey’s post hoc test. (n = 5) (* = p < 0.05, **=p < 0.005, ***=p < 0.001).

The amount of TNF-α in the PFC of WT infected mice significantly (P < 0.005) increased compared to the non-infected control and mice treated with either Diminazine or Iloprost (p < 0.05, Fig. 15A). There were no statistical differences in TNF-α levels in the PFC of Diminazine or Iloprost treated mice compared to the non-infected control (p > 0.05, Fig. 15A). In the HPC, TNF-α levels increased significantly (p < 0.05) in the infected- groups of WT and IL-4Rα┴ mice (p < 0.05, Fig. 15B) compared to their respective non-infected groups. A significant increase in TNF-α levels in the HPC of Diminazine- or Iloprost-treated groups was detected compared to the non-infected mice (P < 0.005) in both the WT and IL-4Rα┴ mice (Fig. 15B).

#### 3.10.2 NF-κB

**Fig. 16:**
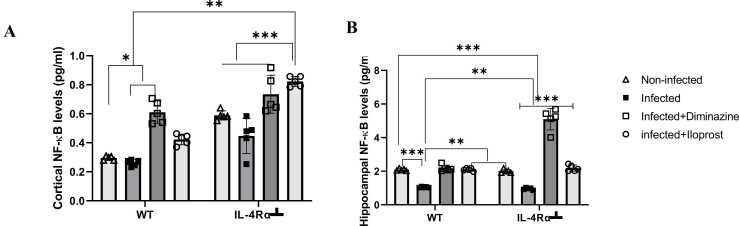
Assessment of NF-kB in the PFC (A) and HPC (B) of mice infected with *T.brucei* and the control mice. Data represented as mean ± SD of independent experiments. Statistical significance was determined using Two-Way ANOVA with Tukey’s post hoc test. (n = 5) (** = p < 0.005, ***=p < 0.001).

Significantly decreased NF-κB levels (p < 0.05) were detected in the PFC of WT-infected mice compared to the non-infected mice, while IL-4Rα┴ infected mice showed decreased NF-κB levels compared to non-infected mice. The treatment groups (i.e Diminazine or Iloprost-treated mice) expressed significantly elevated levels of NF-κB compared to non-infected WT and IL-4Rα┴ mice. This suggests that the treatment potentiates the levels of NF-κB in the PFC and facilitates the elimination of the parasite, unlike in the infected groups, where there were significantly decreased levels of NF-κB (P < 0.005, Fig. 16A). In the HPC, there was a significantly decreased level of NF-κB in infected WT and IL-4Rα┴ mice compared to their non-infected controls. However, there was significantly elevated NF-κB levels (p < 0.005) in the Diminazine-treated mice compared to the non-infected IL-4Rα┴ mice (Fig. 16B). This shows that IL-4Rα┴ mice treated with Diminazine promoted increase of NF-κB in the HPC compared to the Diminazine-treated WT mice. This indicates a dual role, balancing between neuroprotection in acute responses and neurotoxicity during chronic activation, with implications for inflammation, memory, and pathogenesis of trypanosome.

#### 3.10.3 Nitric oxide (NO)

**Fig. 17:**
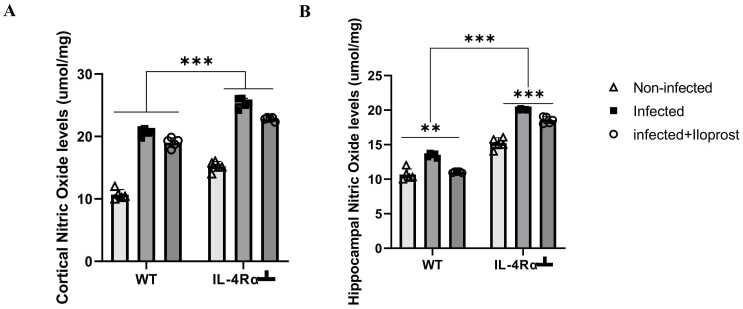
Assessment of Nitric oxide (NO) in the PFC (A) and HPC (B) of mice infected with *T.brucei* and the control mice. Data represented as mean ± SD of independent experiments. Statistical significance was determined using Two-Way ANOVA with Tukey’s post hoc test. (n = 5) (** = p < 0.005, ***=p < 0.001).

A significant increase in NO levels in the PFC of WT-infected mice was observed compared to the non-infected mice, same as the IL-4Rα┴ infected mice (p < 0.001, Fig. 17A). Iloprost treatment was associated with decreased NO levels compared to the infected WT and IL-4Rα┴ mice (Fig. 17A). In the HPC, there was a significant increase of NO levels in WT and IL-4Rα┴ infected mice compared to non-infected mice (p < 0.005, Fig. 17B). Infected WT mice treated with Iloprost showed a significant decrease in NO levels compared to the non-treated WT infected mice (p < 0.005, Fig. 17B). Treatment with Iloprost decreased the levels of NO in both PFC and HPC of WT and IL-4Rα┴mice. The increase was more notable in the HPC of the WT mice.

## 4 Discussion

Possible invasion of the brain parenchyma by *T. brucei* was assessed in mice euthanised at 12dpi. Using PCR amplification of the *ITS* gene produced a 480 bp product from both brain and blood samples of WT and IL-4Rα┴ infected mice. This finding is consistent with the report by Njiru et al. (2011), where members of the *Trypanozoon* subgenus *(T. brucei brucei*, *T. evansi, T. b. rhodesiense* and *T. b. gambiense*) yielded a constant product of approximately 480 bp using *ITS* primers. The presence of this product in the brain tissue suggests that *T. brucei* may have crossed the BBB and begun establishing infection in the CNS at this time point.

In late-stage of trypanosome infection, parasites are known to invade the CNS, leading to neuroinflammation, coma and eventually death if left untreated (MacLean et al., 2013). CXCL10 has been implicated as a key mediator in the pathogenesis of African trypanosomiasis and is considered a candidate marker for late-stage disease (Daniel et al., 2009). In the current study, Iloprost treatment suppressed CXCL10 expression. However, IL-4Rα┴ mice had elevated CXCL10 levels in specific brain regions, potentially due to impaired type 2 immune responses, specifically, the reduced production of IL-4 and IL-13, which are required for the alternative activation of macrophages with anti-inflammatory properties (Stijlemans et al., 2007). These findings indicate that CXCL10 may play a significant role in promoting neuroinflammation in *T. brucei*-infected mice, both in WT and IL-4Rα┴ genotypes, particularly in the PFC and HPC.

At 12 days post-infection (dpi), Amin et al. (2008) reported that the majority of *Trypanosoma brucei* parasites remained confined within cerebral blood vessels, with only occasional presence in the brain parenchyma, marking this stage as the onset of neuroinvasion and an ideal point for assessing early blood-brain barrier (BBB) changes. Immunohistochemical analysis revealed increased BBB permeability, evidenced by enhanced fibrinogen deposition in fresh frozen infected brains and IgG staining restricted to blood vessels, indicating subtle but measurable endothelial leakage that typically precedes extensive parenchymal infiltration. Additionally, there was early upregulation of adhesion molecules ICAM-1 and VCAM-1 on the cerebral vasculature, suggesting activation of the endothelium, which facilitates immune cell and potential parasite transmigration into the CNS. Daniel et al. (2009) further demonstrated elevated expression of CXCL10, a chemokine linked to late-stage neuroinflammation, in key brain regions at this stage, reflecting early CNS immune activation. Adebiyi et al. (2021) also reported evidence of astrocyte activation (GFAP overexpression) and neuronal degeneration (Purkinje cell damage), pointing to the onset of functional neuropathology in the absence of widespread parasite presence in the brain tissue. Collectively, these findings confirm that 12 dpi is a critical transitional phase where *T. brucei*-induced CNS alterations, particularly BBB disruption and neuroinflammatory signalling, are detectable even before significant parenchymal invasion is observed.

The initial body weights of WT-infected mice treated with Iloprost showed a greater improvement over time in body weight compared to the IL-4Rα┴ infected mice treated with Iloprost. This suggests that Iloprost had a more favorable effect on weight recovery in the presence of intact IL-4R signaling. In contrast, the comparatively smaller weight gain observed in the IL-4Rα┴ group may be attributed to impaired type 2 immune response, including diminished IL-4 and IL-13 signalling. This likely led to reduced alternative activation of macrophages, which play a key role in limiting tissue damage and promoting recovery during infection. Weight loss in infected animals is a common marker of disease severity and systemic impact. In mice, it often reflects metabolic alterations, immune activation, and energy depletion caused by infection-induced inflammation. At week 2 post-infection there was a significant weight loss in WT-infected and WT Iloprost-treated infected mice compared to the non-infected mice. This suggests that this stage marks the peak of parasitaemia. *T. brucei* infections are known to induce cachexia and metabolic imbalance due to the release of inflammatory cytokines like TNF-α and IL-6 (Onyilagha and Uzonna, 2019). In the IL-4Rα┴ infected mice, significant weight loss was observed at week 1 post-infection compared to the IL-4Rα┴ non-infected mice. The more severe weight loss in immunocompromised mice can be attributed to their inability to mount an adequate immune response. These mice may experience unchecked trypanosome replication, leading to higher systemic inflammation and greater tissue damage. Similar observations have been documented in infectious models of HAT, where immunosuppressed mice displayed more profound weight loss and disease progression, underscoring the importance of the immune system in mitigating weight loss and systemic effects (Migchelsen et al., 2011, Ortiz-Martínez et al., 2023).

In this study, the white cell counts increased (leukocytosis) in the WT Iloprost-treated mice. This could be due to the vasodilating potential and consequent lymphocytosis observed (Jin et al., 2011). These cells are required for an immunological response against the invading parasite and modulation of allergic inflammatory response respectively (ILRAD, 1990). The report of John et al. (2006) of a similar study using vervet monkeys is consistent with this finding. The present study revealed a significant (p < 0.05) increase in the IL-4Rα┴ group of mice in comparison to the WT mice, though Diminazine treatment in both instances ameliorated the inflammation. Neutrophils are first-responder white blood cells that are mobilized to sites of acute endothelial damage and/or infection (Wilgus et al., 2013). This shows a severe inflammatory response in the IL-4Rα┴ mice. While neutrophils are important in acute inflammation and defense of infections, chronically elevated neutrophil activity can lead to tissue damage (Smith, 1994). Lymphocytes are another type of white blood cell central to the immune response. Lymphocytes are key in the response to infection and are related to the progression of inflammatory and autoimmunity diseases (Brooks et al., 1979, Buseyne et al., 1998). Lymphocytes counts were significantly different in the WT-infected and IL-4Rα┴ infected Iloprost-treated mice only, which suggests that inhibition of the IL-4R causes feedback on the primordial lymphoid cells in moderation of lymphocytosis. Though both WT and IL-4Rα┴ mice showed elevated lymphocyte percentages. However, the Diminazine and Iloprost treatments also mitigated this inflammation in both instances. The neutrophil-to-lymphocyte ratio (NLR) is obtained from complete blood counts (typically 1000 /μl). Because neutrophil and lymphocyte counts are standard in routine blood tests, the NLR represents a cost-effective and easily available but non-specific marker of inflammation. NLR is associated with incidence, morbidity, and mortality in several systemic diseases, including cardiovascular diseases and malignancies (Afari and Bhat, 2016, Küçük et al., 2016, Isaac et al., 2016). More recently, elevated NLR has been associated with schizophrenia and mood disorders (Aydin Sunbul et al., 2016, Bustan et al., 2018, Ivković et al., 2016, Mayda et al., 2016, Mazza et al., 2019, Mazza et al., 2018, Moody and Miller, 2018). The current study used NLR to test if IL-4Rα inhibition adds further to the inflammatory burden in trypanosomiasis and it was seen that IL-4R inhibition promoted inflammation significantly.

The present study investigated the influence of Trypanosomiasis on anxiety-like behaviour in the elevated plus maze in WT and IL-4Rα┴ mice treated with Iloprost. Increase time spent in the open arms was observed for infected WT and IL-4Rα┴ mice that can be attributed mainly to *T. brucei* infection, which promotes anxiolytic exploratory behaviour. In IL-4Rα┴ infected mice, the behavioural manifestation was further increased by pharmacological inhibition of IL-4Rα activity. The combined effects of *T. brucei* infection and inhibition of IL-4Rα activity are further reinforced by the increased number of entries made compared to the non-infected mice (Fig. 5A). Meanwhile, the reverse was the case for exploration of the closed arms (Fig. 5B). Regarding the treatment groups, I can speculate that the treatments with Diminazine or Iloprost prevented trypanosome-induced changes in open-arm entries in the mice. The Iloprost treatment produced anxiolytic-like behaviour, though different to the Diminazine-treated WT and IL-4Rα┴ mice. The WT infected mice exhibited anxiogenic-like behaviour compared to other groups. Both Iloprost and Diminazene treated infected mice spent less time in the open arm, indicating an anxiolytic-like effect compared to the infected non-treated mice. The WT infected were more anxious than the infected IL-4Rα┴ mice in the number of entries into the closed arm, this may indicate that inhibition of IL4-Rα may reverse anxiety-like behaviours (Griebel et al., 1997, Varty et al., 2002). WT Diminazine or Iloprost-treated mice displayed anxiety-like behaviour by a reduction of open-arm exploration, reflected by a decrease in the time spent in the open arms compared to infected mice. Treatment with Iloprost or Diminazine produced anxiolytic effects consistent with previous studies on anxiolytic drugs and neurosteroids like progesterone and allopregnanolone (Engin and Treit, 2007, Fern´andez-Guasti et al., 2001, G´omez et al., 2002, Pellow et al., 1985, Reddy et al., 2005, Wilson et al., 2004). This study found that IL-4Rα inhibition reduced anxiety-like behaviour, while Diminazine treatment increased anxiety-like behaviour in trypanosomiasis. This could suggest that Iloprost and Diminazine played a role in the centre of emotionality, one of which is the PFC and HPC (Woon et al., 2020). These regions work together to regulate fear responses. The PFC sends inhibitory signals to the amygdala (a key player in fear) based on input from the HPC about whether a situation is safe or dangerous (Kredlow et al., 2022).

WT and IL-4Rα┴ Infected mice produced hypoactivity in the open field test reflected by a reduction in the number of crossings, thigmotaxis and rearing time; and an increase in resting time and time spent in the central square. However, mice treated with Iloprost or Diminazene exhibited hyperactivity in the open field test reflected by the increase in the number of crossings in both WT and IL-4Rα┴ mice and a decrease in resting time and time spent in the central square. *T. b. brucei* infection decreased locomotor activity in WT-infected mice and much more in IL-4Rα┴ infected mice, as evidenced by a decline in the total number of lines crossed. This could be because the impact of trypanosome infection on locomotion was further exacerbated by the inhibition of IL-4Rα activity. The hyperactivity identified in the open field test may be related to a potential combined effect of Iloprost and endogenous IL-4Rα during Trypanosomiasis, which together could act on GABA_A_ receptors and disrupt locomotor activity via antagonistic excitation of N-methyl-D-aspartate receptor (NMDAR) via glutamatergic pathways. Also, prostaglandins elicit various biological effects including regulation of smooth muscles, inflammation and immune response (Aliancy et al., 2017). This results in stimulation of soluble Guanylate cyclase (GC) activity and cyclic Guanosine monophosphate (cGMP) formation, thereby leading to muscle relaxation and enhanced activity (Cavet et al., 2014, Muenster et al., 2017). This synergistic potential of GABA_A_ receptor and relaxation of muscle may likely be the mechanism by which this motor activity is exploited. Interestingly, the combined effects of trypanosome infection and compromised immunity on locomotion were mitigated by both Diminazine and Iloprost treatments.

It was observed that there was increased thigmotaxis, an index of anxiety-like behaviours, exhibited by WT-Iloprost treated infected mice which differed from its counterpart IL-4Rα┴ mice. Increased thigmotaxis in an open field is linked to increased anxiety. Hence, decreased thigmotaxis indicates anxiolytic-like behaviour. WT-infected and IL-4Rα┴ infected mice exhibited decreased thigmotaxis and also stayed more at the central square indicating the mice were anxious. This could be due to the *T. brucei* infection. This further emphasizes the reason why the decrease was significantly greater in the IL-4Rα┴ infected mice compared to the WT-infected mice, which could be due to inhibition of IL-4Rα that diminishes anxiety-like behaviour in trypanosomiasis infection. Again, rearing time an index of exploratory activity was more enhanced in the IL-4Rα┴ diminazine-treated mice than the WT mice, an indication that IL-4Rα inhibition improves exploratory activity.

The cerebral cortex of the brain was examined histologically, revealing in infected WT mice, there was necrosis and congestion of cerebral blood vessels, along with cellular infiltration. WT mice treated with Iloprost showed congestion of cerebral blood vessels, while WT mice treated with Diminazine exhibited cellular infiltration in the cerebrum. In contrast, infected IL-4Rα┴ mice displayed severe necrosis of cerebral tissue. IL-4Rα┴ mice treated with Iloprost and Diminazine also showed necrosis of cerebral tissue. This demonstrated that *T. brucei* causes severe brain damage and penetrates the brain tissue. In the present study, *T. brucei* infection caused considerable neuro-histopathological changes in the form of inflammation, haemorrhage, and structural changes in Purkinje cells and congestion of cerebral blood vessels (Black arrows) with cellular infiltration revealed in all groups of WT mice and IL-4Rα┴Iloprost-treated mice. Moreover, the infection-induced a decrease in the number of cells in the Purkinje’s layer. It has been reported that pathological changes in the brain are due to constant irritation caused by the presence of parasites or toxins. In the second stage of the disease, severe and potentially fatal clinical symptoms were reported to cause infiltration and dissemination of *T. brucei* in the CNS (Berlin et al., 2009). Besides, pathogenic brain lesions are reported in both sleeping sickness patients (Schmidt and Bafort, 1987) and in animals infected with *T. brucei* (Keita et al., 1997). The IL-4Rα┴ group experienced severed pathological damage, this could be as a result of deficiency of anti-inflammatory cytokines IL-4 and Il-13 in circulation. However, this study also revealed that neither Diminazene nor Iloprost restored/repaired the damage caused by the parasite. However, Iloprost could improve/neuroprotect the histopathological alteration induced by the parasite and this may be due to the enrichment of anti-apoptotic actions of prostaglandins observable in specific neuronal models. For example, PGA1 which is derived from PGE2 blunts NMDA receptor-mediated neuronal apoptosis by a mechanism involving upregulation of neuroprotective heat shock proteins and inhibition of NF-κB activity (Qin et al., 2001).

The observed changes such as neuroinflammation, necrosis, diminished anxiety-like exploratory behaviour, hypoactivity, cognitive deficit and other neuro-behavioural changes in WT-infected and IL-4Rα┴ infected mice in *T. brucei* infection may have been a result of molecular alteration in gene expression from structures in the brain responsible for their functions. These neuroadaptive changes and alterations in gene expression within brain structures stem from the mesocorticolimbic circuit, especially the PFC (that mediates executive functions) and the HPC (which is involved in context-specific memories) (Lossi et al., 2024). This circuit includes the PFC, HPC and other subcortical structures necessary for identifying and coordinating behavioural and physiological responses to threats (Blanchard and Blanchard, 2008, LeDoux, 2000). This study assessed cognition, memory, and locomotor activity as indicators of the overall functioning of prefrontal and hippocampus regions in IL-4R-inhibited and WT mice.

IL-4Rα mRNA levels were upregulated in the WT-treated group but downregulated in WT-infected and IL-4Rα┴ mice. Decreased IL-4Rα positive macrophage recruitment to a site of injury or infection has been linked to the inability to promote IL-4 signalling via IL-4Rα and impairment in induction of IL-4Rα on microglia is noted as a contributing factor to reduced recovery (Fenn et al., 2014).

Our results revealed that mRNA for TNF-α showed that IL-4Rα-inhibited mice had increased upregulation of this gene, especially in the infected group in both PFC and HPC. However, upon administration of Iloprost and diminazene, it was downregulated. This provides further evidence that this (TNF-α) cytokine is important in the development of inflammation in the CNS. Additionally, TNF-α was assessed using ELISA and a similar result was obtained with increased levels in the IL-4Rα┴ than the WT of both PFC and HPC. Recent work has shown that the administration of TNF-α, IL-lα, and MIP-1 to rabbits can cause inflammation and tissue damage (Ramilo et al., 1990, Saukkonen et al., 1990). VSG-dependent inflammatory responses are mainly triggered by IFNγ-activated macrophages, after recognition of the polymannose carbohydrate core of the VSG glycosylphosphatidylinositol anchor (Harris et al., 2007). This triggers the production of TNF and the expression and shedding of the TNF receptor (Magez et al., 1998). For both *T. brucei* and *T. congolense*, TNF is crucial for peak parasitemia control (Magez et al., 1997, Magez et al., 2004) in conjunction with nitric oxide (NO). Counteracting this inflammatory response in the initial stage of infection appears to be the role of the multiple receptor-like adenylate cyclases of the parasite. Similarly, a released kinesin heavy chain (*Tb*KHC1) can promote myeloid cells to synthesize the anti-inflammatory interleukin 10 (IL-10), thereby stimulating the growth-promoting activity of arginase-1 and conversely inhibiting the antiparasitic activity of the NO synthase (Szempruch et al., 2016). This NO result was revealed where levels of NO WT-infected and IL-4Rα┴ infected mice were significantly elevated and Iloprost intervention decreased the levels of NO. The parasite release of tryptophan-derived indole-3-pyruvate inhibits the production of inflammatory prostaglandins in activated macrophages, which contributes to promoting the survival of both host and parasite (De MuylderG, Daulouède et al., 2013). However, exogenous administration of prostaglandin analogue (Iloprost) in IL-4Rα intact mice potentiates the anti-inflammatory effect of IL-4 and Il-13, that conversely activated macrophage which sustains the survival of host and discourages survival of the parasite, which suppresses pro-inflammatory cells (TNF-α). This T-cell product is a costimulant for B-cell proliferation and can also enhance the expression of class II (Ia) major histocompatibility antigens (Pays et al., 2023). Our results showed that IL-4Ra was upregulated in WT in the treated group but downregulated in IL-4Rα┴ mice. This shows that decreased IL-4Ra positive macrophage recruitment to a site of injury or infection has been linked to the inability to promote IL-4 signalling via IL-4Ra, impairment in induction of IL-4Ra on microglia; a contributing factor to reduced recovery (Fenn et al., 2014). Furthermore, since IL-4Ra signalling has been inhibited during Trypanosoma infection, IL-4 and IL-13 expressions were downregulated in IL-4Rα┴ whereas upregulated in their counterpart WT.

We further probed the involvement of BDNF in the reference memory formation pathway by engaging transcription factor BDNF in the HPC and PFC. Our results revealed steady expression levels of BDNF in the hippocampus and prefrontal cortex of Iloprost-treated mice, though contrary to the wild type, that correlate directly to successful learning for wild-type and IL-4Rα┴ mice. Furthermore, during the novel memory phase of the task of the infected mice, the effects of steady expression levels of BDNF observed in the prefrontal cortex correlate to impaired reference memory. BDNF receptor tropomyosin-related kinase B (TrkB) and activity-regulated cytoskeleton-associated protein (ARC) are known for their interactive importance for spatial memory in the hippocampus (Andero et al., 2014, Gao et al., 2018, Kuipers et al., 2016, Mizuno et al., 2003). Transcription factor c-AMP-responsive element binding protein (CREB) regulates gene expression for both adaptive neuronal responses (Ginty et al., 1994), including BDNF, and complex functions that involve learning and memory (Frank and Greenberg, 1994, Silva et al., 1998). In this study, over- and under- expression of BDNF in the HPC and PFC of WT and IL-4Rα┴ in infected groups results in depression-like behaviours and cognitive impairment which is similar to a report by Cunha et al., 2006, Sakata et al., 2010, Taliaz et al., 2010. Similarly, postmortem and clinical studies have revealed reduced BDNF expression in the brain and peripheral blood of suicide completers and patients with mood disorders, suggesting that reduction in BDNF expression may contribute to these phenotypes (Cunha et al., 2006, Dwivedi et al., 2003, Thompson Ray et al., 2011, Kim et al., 2007). Altered expression of other genes expressed in the brain, which include γ-aminobutyric acid and serotonin transporter, have also been observed to affect behaviour (Lee et al., 2007, Luscher et al., 2011). Together, these studies underscore that altered levels of gene expression in the brain can have a negative impact on behaviour. A major avenue by which stress such as Trypanosoma infection can induce changes to gene expression levels is by altering DNA methylation patterns.

After behavioural phenotyping, we then set out to identify changes in the DNA methylation status of the promoter regions of the above genes in the PFC and HPC. DNA methylation represents an important regulatory mechanism that induces long-term stable changes in gene expression patterns. It is also involved in regulating DNA replication, repair and transposition, and chromatin packaging (Robertson, 2005). These patterns may be determined by factors external to the body, for example, environmental factors (LaSalle, 2011) and may subsequently influence the behaviour of the individual (Phillips and Roth, 2019) and infections such as T. brucei (Militello et al., 2008, Rojas and Galanti, 1990). Our data therefore points to some role of IL-4Rα as one of the key regulators of gene expression changes recruited in the functional activation of neural networks that mediate locomotor, anxiety and reference memory to trypanosome infection effects. IL-4Rα is also known to play a significant physiological role in memory and long-term potentiation (Brombacher et al., 2020). There is compelling evidence that DNA methylation regulates gene transcription necessary for memory function and anxiety (Mikaelsson and Miller 2011). Our investigation indicated IL-4Rα hypermethylation in the PFC of mice, suggesting transcriptional silencing of memory-linked genes regulated by IL-4Rα in the PFC of WT, which contrasted with the IL-4Rα┴. This is in line with previous findings that established that long-term potentiation is unaltered in Crem is enhanced by the IL-4Rα channel (Maldonado et al., 1999). Ajonijebu et al. (2017) reported that observed molecular changes in Crem and fosb promoter, thus demonstrating that cocaine consumption did not alter recognition memory but rather promoted novelty-seeking behaviour. Our result revealed that BDNF promoter was hypomethylated in both PFC and HPC of WT. This demonstrates that neuronal depolarization resulted in hypomethylation within the transcriptional regulatory region of the brain-derived neurotrophic factor (BDNF) gene, along with a corresponding increase in BDNF mRNA expression which is similar to the report by Martinowich et al. (1998). This seminal finding pointed towards the dynamic regulation of DNA methylation as a potential mediator of activity-dependent transcriptional regulation within the CNS. The evidence of reduced methylation, and increased BDNF gene expression in response to neuronal stimulation, was consistent with a working model implicating transcriptional regulation as being permissive for synaptic plasticity and memory (Abel and Kandel, 1998, Day and Sweatt, 2010, Kandel, 2001, Pittenger and Kandel, 1998). In accordance with this model, HDAC-inhibitors, which were generally thought to promote gene transcription, were found to enhance synaptic plasticity (Levenson et al., 2004). Therefore, it was reasoned that an intervention believed to have a positive effect on gene transcription, such as blocking DNA methylation with DNMT inhibitors, would ultimately lead to an enhancement in synaptic plasticity. A study involving hippocampus slices bath-treated with the non-specific DNMT-inhibitor zebularine (Zeb) detected an acute (40 min) decrease in DNA methylation at the promoters of two genes whose expression is positively correlated with memory formation: *Reelin* (*R*ln ) and *Brain-derived neurotrophic factor exon 1* (*Bdnfex1)* (Levenson et al., 2006, Levenson and Sweatt, 2008, Weeber et al., 2002). Yet, a perplexing result revealed that pre-treatment with two structurally distinct DNMT inhibitions, Zeb and 5-aza-2′-deoxycytidine (5-Aza), led to a diminution in long-term potentiation (LTP), the cellular correlate of memory (Bliss and Collingridge, 1993, Levenson et al., 2006). Although for the first time this study revealed evidence of dynamic regulation of DNA methylation within the PFC and HPC of trypanosome-infected mice, the results were seemingly opposing to the working model in which the suppression of gene expression was disruptive towards memory formation.

## 5 Conclusion

This study provides comprehensive insights into the pathophysiological and neurobehavioral effects of *Trypanosoma brucei* infection in both WT and IL-4Rα┴ mice. The findings suggest that *T. brucei* successfully invades the central nervous system, evidenced by PCR detection of the parasite in both the brain and blood. CXCL10 emerged as a key mediator of neuroinflammation, particularly in IL-4Rα┴ mice, highlighting its role in disease progression. The suppression of CXCL10 by Iloprost and the differential immune responses between WT and IL-4Rα┴ mice emphasize the importance of IL-4Rα signalling in controlling inflammation and immune homeostasis.

Infected mice demonstrated significant weight loss, leukocytosis, altered neutrophil-to-lymphocyte ratios, and pronounced neurobehavioral changes, including increased anxiety-like behaviours and hypoactivity. These effects were more severe in IL-4Rα┴ mice, suggesting that IL-4Rα plays a protective role in modulating immune responses and mitigating disease severity. Histopathological analyses revealed severe neurodegeneration, necrosis, and inflammation in the brain, particularly in IL-4Rα┴ mice, underscoring the detrimental impact of impaired anti-inflammatory signalling.

Pharmacological interventions with Iloprost demonstrated efficacy in reducing neuroinflammation and some neurobehavioral abnormalities. Iloprost exhibited potential neuroprotective effects as well, likely through its anti-inflammatory and anti-apoptotic actions, although it did not fully reverse histopathological damage. Moreover, IL-4Rα inhibition was associated with downregulation of anti-inflammatory cytokines, upregulation of TNF-α and nitric oxide, and altered BDNF expression, collectively contributing to cognitive dysfunction and exacerbated neuroinflammation and anxiety-like behaviours.

## 6 Recommendations

1. Therapeutic Targeting of CXCL10 and TNF-α: Since CXCL10 and TNF-α play significant roles in promoting neuroinflammation and disease progression, targeted therapies aimed at modulating these cytokines may improve outcomes in African trypanosomiasis.
2. Enhancing IL-4Rα Signalling: Preservation or pharmacological enhancement of IL-4Rα activity could be a potential therapeutic strategy to boost anti-inflammatory responses, reduce neuroinflammation, and improve recovery in late-stage trypanosomiasis.
3. Combination Therapy with Iloprost and Antiparasitic Drugs: The partial neuroprotective effects of Iloprost suggest it may be a valuable adjunct therapy when combined with trypanocidal drugs like Diminazine to alleviate neuroinflammation and improve neurobehavioral outcomes.
4. Further Investigation of Neurotrophic Factors: Altered BDNF expression in infected mice points to a role in cognitive deficits. Future studies should explore the therapeutic potential of BDNF modulation or TrkB agonists to address cognitive impairments associated with trypanosome infections.

## CRediT authorship contribution statement

**Olaolu OS:** Writing – original draft, Methodology, Investigation, Formal analysis, Validation and Data curation. **Davids H:** Reviewing, editing and supervision. **Dealtry GB:** Writing- editing, reviewing and supervision. **Abubakar AS:** Methodology. **Obishakin ET:** Methodology and Investigation. **Iliyasu B:** Methodology and Validation. **Azeez IA:** Writing – review & editing, Methodology, Data curation. **Ajonijebu DC:** Writing- editing & reviewing, Supervision, Project administration, Methodology, Funding acquisition, Conceptualization.

## Declaration of Competing Interest

I am writing to declare that the authors of this manuscript have complied with the ethical standards and have no competing interests exist.
